# Associations of fatigue to work-related stress, mental and physical health in an employed community sample

**DOI:** 10.1186/s12888-017-1237-y

**Published:** 2017-05-05

**Authors:** D. M. Rose, A. Seidler, M. Nübling, U. Latza, E. Brähler, E. M. Klein, J. Wiltink, M. Michal, S. Nickels, P. S. Wild, J. König, M. Claus, S. Letzel, M. E. Beutel

**Affiliations:** 1 Institute of Teachers’ Health, University Medical Center of the Johannes Gutenberg University of Mainz, Mainz, Germany; 2 Institute and Policlinic of Occupational and Social Medicine, Faculty of Medicine, TU Dresden, Dresden, Germany; 3 FFAW, Freiburg Research Centre for Occupational Sciences, Freiburg, Germany; 4 Federal Institute for Occupational Safety and Health (BAuA), Berlin, Germany; 5 Department of Psychosomatic Medicine and Psychotherapy, University Medical Center of the Johannes Gutenberg University of Mainz, Mainz, Germany; 6 Department of Ophthalmology, University Medical Center of the Johannes Gutenberg University of Mainz, Mainz, Germany; 7 Department of Medicine 2, Preventive Cardiology and Preventive Medicine, University Medical Center of the Johannes Gutenberg University of Mainz, Mainz, Germany; 8 German Center for Cardiovascular Research (DZHK), partner site RhineMain, Berlin, Germany; 9 Center for Thrombosis and Hemostasis (CTH), University Medical Center of the Johannes Gutenberg University of Mainz, Mainz, Germany; 10 Institute of Medical Biostatistics, Epidemiology and Informatics (IMBEI), University Medical Center of the Johannes Gutenberg University of Mainz, Mainz, Germany; 11 Institute of Occupational, Social and Environmental Health, University Medical Center of the Johannes Gutenberg University of Mainz, Mainz, Germany

**Keywords:** Fatigue, Depression, Work-related stressors, Allostatic load, Health behavior

## Abstract

**Background:**

While work-related fatigue has become an issue of concern among European employees, the relationship between fatigue, depression and work-related stressors is far from clear. The purposes of this study were (1) to determine the associations of fatigue with work-related stressors, severe medical disease, health behavior and depression in the working population and (2) to determine the unique impact of work-related stressors on fatigue.

**Methods:**

We used cross-sectional data of *N* = 7,930 working participants enrolled in the Gutenberg Health Study (GHS) from 2007 to 2012 filled out the Personal Burnout Scale (PBS) of the Copenhagen Psychosocial Questionnaire (COPSOQ), the PHQ-9, and a list of work-related stressors.

**Results:**

A total of 27.5% reported increased fatigue, esp. women, younger persons with a lower social status and income, smokers, severely medically ill, previously and currently depressed participants. Fatigue was consistently associated with severe medical disease, health behavior and depression, which need to be taken into account as potential confounders when analyzing its relationship to work-related strains. Depression was consistently associated with work-related stressors. However, after statistically partialling out depression, fatigue was still significantly associated with work-related stress.

**Conclusions:**

Fatigue as an indicator of allostatic load is consistently associated with work-related stressors such as work overload after controlling for depression. The brief Personal Burn-out Scale is suitable for assessing work-related fatigue in the general population.

## Background

Fatigue has been defined as the subjective experience of tiredness or lack of energy [1]. Normal tiredness is usually not experienced as an unpleasant state, since it can be remedied by rest and sleep. Fatigue, however, has an unpleasant quality; it is not necessarily related to exertion and is not easily or fully restored by rest or sleep [2]. Fatigue has been described in the context of work-related strains, but also in relation to chronic medical disease [2]. Work-related fatigue has become an issue of concern among European employees resulting from prolonged work-related stress [3]. Absenteeism from work [4] and ill mental and physical health have been described as consequences [5]. The Personal Burnout Scale (PBS) of the Copenhagen Psychosocial Questionnaire (COPSOQ) is a brief and reliable scale of 6 items assessing tiredness and exhaustion as indicators of work-strains. Indeed, fatigue has been consistently used as a core criterion of burnout along with cynism and reduced work efficacy [6]. Despite its popularity, however, research on burnout is hampered by the lack of final consensus for its definition [7] or binding diagnostic criteria for its assessment [8]. In a broad sense, burnout refers to “a negative work-related state of mind that is preceded by chronic work stress” ([9], p. 1). Burnout is no defined medical diagnosis. The ICD-10 only has the option to code Z73.0 as an additional criterion to denote problems coping with demands in life, e.g. burnout.

The Personal Burnout Scale, as defined by the COPSOQ, has been found strongly associated with work-related strains, especially work-privacy conflict, reduced possibilities of development, emotional demands, job insecurity and little freedom at work [10]. Numerous studies have associated burnout with impaired health behavior (e.g. physical inactivity [11], overeating [12]), medically certified sickness absences in the general population [13] and in specific professional groups (e.g. the health sector). A recent large-scale analysis of sickness leave data in Germany (including almost 85% of members of the statutory health insurance), have shown a strong increase of medically certified burnout, from 0.7 days off from work in 2004 to 9.1 days per 100 members of the health insurance companies in 2011. Burnout has also been associated with multiple physical illnesses [1, 14].

Allostasis refers to the adaptation to the social and physical environment [15]. The cost of adaptation to adverse conditions has been termed allostatic load [16]. The allostatic load by prolonged and unsuccessful attempts at adaptation may lead to impaired immunity, metabolic syndrome, atherosclerosis, and even damage to the brain such as atrophy of nerve cells [17]. Ganster & Rosen (2013) proposed that allostatic load processes may fruitfully explain the effects of workplace experiences on mental and physical well-being [18]. Accordingly, a recent cross-sectional study by Hintsa et al. (2014) investigating three dimensions of burnout found that exhaustion, cynism and decreased efficacy each predicted allostatic load (measured by a composite index of a metabolic syndrome and inflammation) [9]. These associations, however, were no more significant after including depression which explained about 60% of the association.

Burnout has been shown to be related to depression [14], a major health problem among working populations leading to increasing and prolonged sickness absences [19]. This raises the issue of differential diagnosis [14]. However, few studies have examined the relationship between depression and burnout. In a prospective study with Finnish dentists, Ahola & Hakanen (2007) found that burnout at baseline was a predictor of depression at the 3-year follow-up [1]. There was a strong effect of job strain on burnout, which remained significant after adjustment for depression. Armon et al. (2014) found that burnout and chronic medical illness predicted depression in employed men and women [20].

The purposes of this study were (1) to determine the associations of fatigue with work-related stressors, severe medical disease, health behavior and depression in the working population and (2) to determine the unique impact of work-related stressors on fatigue.

## Methods

### Procedure and study sample

We investigated cross-sectional data of *N* = 7.930 working participants (6,204 full-time and 1,726 part-time employed) enrolled in the Gutenberg Health Study (GHS) from 2007 to 2012 who had received the Copenhagen Psychosocial Questionnaire (COPSOQ). The GHS is a population-based, prospective, observational single-center cohort study in the Rhine-Main-Region in western Mid-Germany. The study protocol and study documents were approved by the local ethics committee of the Medical Chamber of Rhineland-Palatinate, Germany (reference no. 837.020.07; original vote: 22.3.2007, latest update: 20.10.2015) and by the local and federal data safety commissioners. The primary aim of the study is to evaluate and improve cardiovascular risk stratification. The sample was drawn randomly from the local registry in the city of Mainz and the district of Mainz-Bingen. The sample was stratified 1:1 for sex and residence and in equal strata for decades of age. Inclusion criteria were age 35 to 74 years and written informed consent. Persons with insufficient knowledge of German language, or those who reported that they were not able to visit the study center on their own (due to their physical and/or mental condition) were excluded. The response rate^1^ was 60.3% for the first 5.000 participants. Due to the ongoing recruitment of the GHS, which is conducted in waves, a final statement concerning the response rate cannot be made at this time. The design and the rationale of the Gutenberg Health Study (GHS) have already been described in detail elsewhere [21].

### Materials and assessment

The 5-h baseline-examination in the study center comprised evaluation of prevalent classical cardiovascular risk factors and clinical variables, a computer-assisted personal interview, laboratory examinations from a venous blood sample, blood pressure and anthropometric measurements. In general, all examinations were taken out according to standard operating procedures (SOPs) by certified medical technical assistants.

### Measures

The Personal Burnout Scale (PBS) is part of the Copenhagen Psychosocial Questionnaire with 6 Items assessing physical and mental exhaustion, independently from work. It assesses the frequency of the following items („How often do you feel …“: tired, physically exhausted, emotionally exhausted, unable to go on, weak and prone to illness). Ratings are done on a 5-point scale 1 = never/almost never, 2 = rarely, 3 = occasionally, 4 = often, 5 = always (COPSOQ [22]). Data were transformed to a metric scale (1 = 0; 2 = 25 to 5 = 100) („high burnout^2^“). The scale is reliable (Cronbach alpha of the German version = 0.91 [23]); a mean score ≥ 50 was considered evidence for the presence of fatigue [24].

In order to cover a broad range, work-related stressors (work overload, piece/shift work, insufficient vacation, frequent conflicts with supervisors or colleagues and unemployment of the partner) were assessed by single items using 5-point scales (0 = no, does not apply, 1 = yes it applies, but it does not stress me, 2 = yes, it applies, and it stresses me slightly (3 = moderately, 4 = strongly). Items were recoded combining 0 and 1 (no strain or no stress); 2 = slightly, 3 = moderately and 4 = severely stressed.

Depression was measured by the Patient Health Questionnaire (PHQ-9); caseness of depression was defined by a score ≥ 10 with a sensitivity of 81% and a specificity of 82% for depressive disorder [25]. Further depressive symptoms can be classified as “minimal” (score 5 to 9), “mild” (score 10 to 14), moderately severe (score 15 to 19) and severe (score ≥ 20). The somatic-affective and cognitive-affective dimensions of depression were defined according to prior studies [26]. Four PHQ-9 items related to problems with sleep, fatigability, appetite, and psychomotor agitation/retardation were classified as somatic-affective symptoms, whereas 5 items, related to lack of interest, depressed mood, negative feelings about self, concentration problems and suicidal ideation, were classified as cognitive-affective symptoms of depression [26].

### Computer-assisted personal interview

During the computer-assisted personal interview, participants were asked whether they had ever received a definite diagnosis of any depressive disorder by a physician (medical history of lifetime diagnosis of any depressive disorder, medical history of depression). Severe medical disease was defined by the presence of coronary heart disease, myocardial infarction, stroke, peripheral artery disease, heart failure, diabetes, cancer, COPD, rheumatic, chronic kidney or liver disease. Diabetes was defined in individuals with a definite diagnosis of diabetes by a physician or a blood glucose level of at least 126 mg/dl in the baseline examination after an overnight fast of at least 8 h or a blood glucose level of at least 200 mg/dl after a fasting period of less than 8 h. The presence of coronary heart disease was assessed by the question: ‘Were you diagnosed with a stenosis of your coronary vessels?’ Other chronic medical diseases were assessed correspondingly. Cardiovascular risk factors were defined as follows: Smoking was dichotomized into nonsmokers (never smoker and ex-smoker) and current smokers (occasional smoker, i.e. <1 cigarette per day, and smoker, i.e. > 1 cigarette per day). Obesity was defined as a BMI of at least 30 kg/m^2^. Unhealthy alcohol intake was defined as habitual alcohol intake of more than 20 g per day for men and more than 10 g per day for women.

The socioeconomic status (SES) was defined according to Lampert and Kroll’s (2009) scores of SES with a range from 3 to 21 (3 indicates the lowest SES and 21 the highest SES) [27].

### Statistical analysis

Data are presented as numbers/percentage, mean (and 1.96-fold standard deviation) or median (and 1st, 3rd quartile) as appropriate. We performed non-parametric and parametric tests as appropriate to compare participants with and without fatigue. In order to identify determinants of fatigue, we computed separate linear regression models with fatigue as the dependent variable. For each of a set of potentially explanatory variables we fitted a series of linear models including that variable and successively more variables for adjustment. Unadjusted effects and all increasingly adjusted effects are reported. Models were pre-specified in a statistical analysis plan; no data-driven model selection procedures have been applied. In a stepwise manner, we adjusted for age, sex and SES, work-related strains, medical disease, health behavior and depression. In face of small proportions of missing values and a large sample size we preferred to perform complete case analysis with respect to set of variables of each fitted model. We reported the number of cases for each model fit.

To determine relations between work-related strains, fatigue and depression we computed Pearson partial correlation coefficients partialling out depression, respectively fatigue from the associations with work-related strains. The difference of the size of partial correlations was determined by Steiger’s Z test [28]. P-values are given for descriptive reasons only and should be interpreted with caution and in connection with effect estimates. All *p*-values correspond to 2-tailed tests; the levels of significance was set at *p* < .05. Statistical analysis was carried out using IBM SPSS Statistics 20 (IBM, Chicago, IL).

## Results

### Fatigue in the general population

A total of 27.5% of the sample fulfilled the criteria for fatigue. Table 1 presents the sample comparing participants without and with fatigue.

**Table 1:** Characteristics of participants with fatigue and control subjects of the German population-based Gutenberg Health Study (GHS), 2007–2012 (*N* = 7,930)

		Fatigue (*n* = 2,184)		No Fatigue (*n* = 5,746)		*p*-value (*χ* ^2^-Test/*t*-Test)
		*n*	%	*n*	%	
Sex	Male	918	20.9	3,475	79.1	*p* < 0.0001
	Female	1,266	35.8	2,271	64.2	
Education	Elementary	606	28.4	1,526	71.6	n.s.
	10^th^ grade	543	28.9	1,337	71.1	
	High school	1,012	26.2	2,845	73.8	
	Other	23	39.0	36	61.0	
Vocational training	Apprenticeship	979	29.9	2,290	70.1	*p* < 0.05
	Technical school	319	25.9	913	74.1	
	University	740	25.1	2,207	74.9	
	Other/none	144	30.8	327	69.2	
Depression (PHQ-9 ≥ 10)	*n* = 630; 7.9%	555	88.1	75	11.9	*p* < 0.0001
Medical history of depression	*n* = 797; 10.1%	480	60.2	317	39.8	*p* < 0.0001
Severe medical disease^a^	*n* = 1,465; 22.7%	501	34.2	964	65.8	*p* < 0.0001
Current Smoking	*n* = 1,884; 23.8%	580	30.8	1,304	69.2	*p* < 0.0001
Alcohol abuse	*n* = 203; 2.6%	62	30.5	141	69.5	
		Mean	SD	Mean	SD	
Age		47.7	7.3	48.5	7.6	*p* < 0.0001
SES		13.6	4.2	14.4	4.2	*p* < 0.0001
PHQ-9 score (Depression)		7.2	4.1	3.0	2.3	*p* < 0.0001
Weekly working hours		40.3	13.4	41.2	12.9	*p* < 0.01
Monthly net income household		3,565.5	2,216.2	4,132.2	2,844.2	*p* < 0.0001
Work overload		2.41	1.37	1.44	1.31	*p* < 0.005
Frequent overtime hours		1.76	1.49	1.28	1.20	*p* < 0.0001
Piece work		0.40	1.04	0.26	0.76	*p* < 0.0001
Shift work		0.15	0.68	0.07	0.40	*p* < 0.0001
Insufficient vacation or leisure time		1.51	1.55	0.74	1.11	*p* < 0.0001
Frequent conflicts with boss		0.91	1.40	0.40	0.95	*p* < 0.0001
Frequent conflicts with colleagues		0.83	1.27	0.41	0.88	*p* < 0.0001
Partner unemployed		0.19	0.75	0.12	0.56	*p* < 0.0001
Cigarettes per day	*n* = 1,727	14.38	10.19	12.97	9.31	*p* < 0.005
BMI		27.1	5.44	26.8	4.68	*p* < 0.01

^a^Severe medical disease 0/1 = CHD or MI or Stroke or PAD or HF or Diabetes or Cancer or COPD or rheumatic disease; or chronic kidney or liver disease

Among respondents, the proportion of women reporting fatigue (35.8%) was higher than among men (20.9%). Fatigued participants were slightly younger, had lower vocational training, socioeconomic status and reported less working hours per week and had a lower income. Concerning health behavior, their BMI was higher, and they smoked more frequently and more intensively, and the rate of severe medical disease was higher. Work-related strains such as work-overload, piece, shift work, insufficient leisure time and conflicts at the workplace were also consistently higher, and also there were higher rates of partner unemployment.

### Predictors of fatigue

Table 2 determines the associations of fatigue with the predictors from Table 1.

**Table 2:** Prediction of fatigue (PBS) based on demographic data, work-related strains, somatic disease, health behavior and depression in the German population-based Gutenberg Health Study (GHS)

	Model1				Model 2				Model 3				Model 4				Model 5			
	N	Coeff.	SE ^p value^	Std. Est	N	Coeff.	SE ^p value^	Std. Est	N	Coeff.	SE ^p value^	Std. Est	N	Coeff.	SE ^p value^	Std. Est	N	Coeff.	SE ^p value^	Std. Est
Predictors																				
Sex	7930	7.47	0.39^<.0001^	0.21																
Age	7930	−0.14	0.03^<.0001^	−0.06																
SES	7928	−0.45	0.05^<.0001^	−0.11																
Health behavior																				
Cigarettes/day	7930	0.16	0.03^<.0001^	0.07	7928	0.15	0.03^<.0001^	0.06												
BMI [%]	7928	0.14	0.04^0.0003^	0.04	7926	0.23	0.04^<.0001^	0.07												
Alcohol abuse	7928	0.32	1.24^0.7979^	0.00	7926	1.38	1.21^0.2551^	0.01												
Severe med disease	7930	4.05	0.50^<.0001^	0.09	7928	4.26	0.50^<.0001^	0.09	7924	3.94	0.50^<.0001^	0.09								
Depression																				
Depression score >9	7920	28.44	0.67^<.0001^	0.43	7918	27.28	0.66^<.0001^	0.41	7914	27.04	0.66^<.0001^	0.41	7914	26.82	0.66^<.0001^	0.41				
Depression score	7920	3.29	0.04^<.0001^	0.65	7918	3.20	0.04^<.0001^	0.63	7914	3.19	0.04^<.0001^	0.63	7914	3.18	0.04^<.0001^	0.63				
Depression somatic	7918	3.50	0.09^<.0001^	0.40	7916	3.29	0.09^<.0001^	0.37	7912	3.27	0.10^<.0001^	0.37	7912	3.23	0.10^<.0001^	0.37				
Depression cognitive	7918	3.07	0.10^<.0001^	0.33	7916	3.10	0.10^<.0001^	0.34	7912	3.12	0.10^<.0001^	0.34	7912	3.12	0.10^<.0001^	0.34				
Medical history of depression	7919	16.16	0.63^<.0001^	0.28	7917	14.70	0.62^<.0001^	0.25	7913	14.52	0.62^<.0001^	0.25	7913	14.30	0.62^<.0001^	0.25				
Work-related stressors																				
Work overload	7867	4.96	0.13^<.0001^	0.40	7865	5.15	0.12^<.0001^	0.41	7861	5.12	0.12^<.0001^	0.41	7861	5.09	0.12^<.0001^	0.41	7842	3.06	0.10^<.0001^	0.24
Frequent overtime hours	7895	2.95	0.15^<.0001^	0.22	7893	3.43	0.14^<.0001^	0.26	7889	3.36	0.14^<.0001^	0.25	7889	3.35	0.14^<.0001^	0.25	7869	2.12	0.11^<.0001^	0.16
Shift work	7880	2.02	0.23^<.0001^	0.10	7878	2.07	0.23^<.0001^	0.10	7874	2.02	0.23^<.0001^	0.10	7874	2.02	0.23^<.0001^	0.10	7854	0.96	0.18^<.0001^	0.05
Piece work	7869	3.03	0.39^<.0001^	0.09	7867	3.00	0.39^<.0001^	0.09	7863	2.93	0.39^<.0001^	0.08	7863	2.91	0.39^<.0001^	0.08	7843	1.30	0.30^<.0001^	0.04
Insufficient vacation or leisure time	7883	4.18	0.14^<.0001^	0.31	7881	4.35	0.14^<.0001^	0.32	7877	4.29	0.14^<.0001^	0.32	7877	4.28	0.14^<.0001^	0.32	7858	2.26	0.12^<.0001^	0.17
Frequent conflicts with boss	7890	3.89	0.17^<.0001^	0.25	7888	3.78	0.17^<.0001^	0.24	7884	3.75	0.17^<.0001^	0.24	7884	3.68	0.17^<.0001^	0.23	7864	1.34	0.14^<.0001^	0.09
Frequent conflicts with colleagues	7910	3.84	0.19^<.0001^	0.22	7908	3.64	0.18^<.0001^	0.21	7904	3.61	0.18^<.0001^	0.21	7904	3.57	0.18^<.0001^	0.21	7884	1.38	0.15^<.0001^	0.08
Partner unemployed	7886	1.79	0.32^<.0001^	0.06	7884	1.28	0.31^<.0001^	0.05	7880	1.23	0.31^<.0001^	0.04	7880	1.20	0.31^0.0001^	0.04	7860	0.22	0.24^0.3539^	0.01

*Note*. Linear regression: model 1 (crude); model 2 adjusted for sex, age and social status; model 3 additionally adjusted for health behavior; model 4 additionally adjusted for somatic disease; model 5 additionally adjusted for depression (somatic, cognitive and history). *Coeff* regression coefficient, *SE* standard error, *Std. Est.* standardized regression coefficient

As Table 2 shows, work related stressors were associated with fatigue in a univariate model without any adjustment and in multivariable models after adjusting for social variables (female sex, lower age, lower SES), work-related strains, severe medical disease, and adverse health behaviors (smoking, higher BMI, but not alcohol abuse). The same applied to the presence of current and previous depression, as well as somatic and cognitive symptoms of depression, all work-related stressors and partner unemployment. Even after controlling for all variables, work-related stressors (but not partner unemployment) remained statistically predictive after controlling for social variables, health behavior, medical disease and depression.

### Correlations between work-related strains, fatigue and depression

Table 3 presents the correlations between work-related strains, fatigue and depression.

**Table 3:** Partial correlations between work-related strains, fatigue and depression (*N* = 7.673) in the German population-based Gutenberg Health Study (GHS)

	Fatigue (PBS) ^a^	Depression (PHQ-9) ^a^	PBS partial PHQ-9	PHQ-9 partial PBS	Steiger’s Z-Test
Work overload	0.42 < .0001	0.30 < .0001	0.32 < .0001	0.04 0.0005	Z = 13.97; *p* < 0.001
Frequent overtime hours	0.26 < .0001	0.16 < .0001	0.21 < .0001	−0.01 0.3397	Z = 10.65; *p* < 0.001
Shift work	0.09 < .0001	0.08 < .0001	0.05 < .0001	0.03 0.0143	Z = 1.30; *p* = 0.193
Piece-rate work	0.08 < .0001	0.08 < .0001	0.04 0.0002	0.03 0.0051	Z = 0.51; *p* = 0.610
Too little vacation or leisure time	0.33 < .0001	0.26 < .0001	0.21 < .0001	0.08 < .0001	Z = 6.83; *p* < 0.001
Frequent conflicts with boss	0.25 < .0001	0.25 < .0001	0.11 < .0001	0.13 < .0001	Z = −0.58; *p* = 0.562
Frequent conflicts with colleagues	0.22 < .0001	0.21 < .0001	0.11 < .0001	0.10 < .0001	Z = 0.43; *p* = 0.668
Partner unemployed	0.04 < .0001	0.06 < .0001	0.01 0.3654	0.04 0.0010	Z = −1.32; *p* = 0.185

^a^ Pearson Partial Correlation Coefficients, Prob > |r| under H0: Partial Rho = 0 partialized with respect to age gender SES only. Columns 3–5 partialized with respect to age, gender, SES, and the indicated construct (DP, CBI and WRS respectively). PBS partial (work related stress items) 0.57; <.0001; Lee & Preacher (2013) [35]

As the table shows, fatigue is most strongly associated with work overload and with lack of vacation or leisure time and frequent overtime hours, and also with frequent conflicts with boss or colleagues. Small and significant associations were found with shift work, piece rate work, and with the unemployment of the partner. Depression was also most strongly correlated with work overload, too little vacation, followed by conflicts with boss or colleagues. All other variables showed small correlations with depression.

When partialling out depression, fatigue was highly significantly associated with all work-related strains. When partialling out fatigue, depression was no longer associated with frequent overtime hours. All other associations between depression work-related stressors and also with partner unemployment became quite small, but remained significant. However, partial correlations between fatigue and work-related stressors were higher compared to depression (Steiger Z-test) regarding work overload, frequent overtime hours and too little vacation or leisure time.

## Discussion

As in other studies (e.g. [29]), there was a high overall proportion of fatigue in the general population, affecting one in four participants. The proportion of fatigue was increased in women vs. men, persons with lower vocational training, socioeconomic status and income and those with shorter working hours per week.

Fatigue was consistently associated with vocational strains, work overload, frequent overtime hours, shift and piece-rate work, too little vacation and leisure time and frequent conflicts with boss or colleagues.

Overall, fatigue was also associated with the presence of somatic disease, and with adverse health behavior (particularly smoking, overweight), current and previous depression. After controlling for social factors, health behavior, current and previous depression, the relationship between fatigue and work-related strains remained significant.

Our findings support that fatigue as one of the crucial indicators of burn-out is consistently associated with work-related stressors such as work overload, frequent overtime hours and too little vacation or leisure time even after controlling for depression. Other strains such as frequent conflicts with boss or with colleagues, however, are associated both with fatigue and with depression. Our findings demonstrate the multifaceted nature of work-related fatigue. Strong associations with sex, social disadvantage (lower status, training, income, partner unemployment) and adverse health conditions can be seen as indicators for vulnerability factors for burnout.

As in previous studies, unhealthy lifestyle behavior, as evidenced by heavy smoking and obesity may be seen as risk factors increasing fatigue and promoting further negative consequences for mental and physical health [30]. Even though there is considerable overlap, fatigue as an indicator of burnout cannot be reduced to depression; there is rather a reciprocal influence between fatigue and depression as specified by Ahola & Hakanen (2007) [1]. Findings are consistent with the concept of allostatic load: as the body adapts to work-related stressors, fatigue arises, particularly when allostatic load accumulates as social disadvantage, somatic and mental disorders are additionally present. Interestingly, a lack of non-work recreation (too little vacation or leisure time, overtime hours) which have been identified as buffering the effects of work-related stresses is another determinant of fatigue [31]. As postulated by McEwen & Seeman (1999), an unhealthy lifestyle behavior, particularly heavy smoking and obesity are additional risk factors [32]. These complex interactions between mental and physical factors makes it understandable, why participants do not easily recover from fatigue, respectively why fatigue may lead to depression in some (but not all) cases [33].

### Limitations

While this is a large and representative population-based data set, conclusions are limited by the cross-sectional nature of the study. Causal inferences cannot be drawn. I.e., it cannot be precluded that there is a temporal sequence with chronic work overload leading to fatigue which then turns into depression. However, some of the correlations (esp. related to depression) are quite small. The assessment of burnout has been limited to fatigue as defined in the Personal Burnout Scale. We cannot preclude common method bias, as we assessed independent and dependent variables by self-report [34]. Clearly, prospective studies are needed that focus more on the specific aspects of fatigue and related work-related stressors as indicators of burnout. We expect to gain more conclusive data from the 5-year follow-up investigations of our study sample.

## Conclusions

Fatigue as one of the crucial indicators of burn-out is consistently associated with work-related stressors in the context of an increased allostatic load. Associations remain after controlling for depression. The Personal Burn-out Scale is a brief scale suitable for assessing work-related fatigue in the general population.

## Abbreviations

BMI: Body-mass-index
COPD: Chronic obstructive pulmonary disease
COPSOQ: Copenhagen psychosocial questionnaire
GHS: Gutenberg health study
ICD: International statistical classification of diseases and related health problems
PBS: Personal burnout scale
PHQ: Patient health questionnaire
SES: Socioeconomic status
SOP: Standard operating procedures

## Footnotes

1 defined as the recruitment efficacy proportion, i.e. the number of persons with participation in or appointment for the baseline examination divided by the sum of number of persons with participation in or appointment for the baseline examination plus those with refusal and those who were not contactable

2
http://www.arbejdsmiljoforskning.dk/da/projekter/puma/saadan-maales-udbraendthed/cbi-in-english
